# Brighter NIR Bioluminescence System for Mammalian Cell Bioimaging Based on Engineered Railroadworm Luciferase and 6′-Aminoluciferin Analogues

**DOI:** 10.1021/cbmi.5c00163

**Published:** 2026-01-14

**Authors:** Gabriel F. Pelentir, Vanessa R. Bevilaqua, Michio Kakiuchi, Takashi Hirano, Vadim R. Viviani

**Affiliations:** † Graduate Program of Biotechnology, 67828 Federal University of São Carlos (UFSCar), São Carlos 13565-905, SP, Brazil; ‡ Biomaterials Laboratory, Medical and Health Sciences Faculty, Pontifical University Catholic of São Paulo (PUC-SP), Sorocaba 18060-030, SP, Brazil; § Department of Engineering Science, Graduate School of Informatics and Engineering, The University of Electro-Communications, Chofu, Tokyo 182-8585, Japan; ▼ Department of Physics, Chemistry and Mathematics (DFQM), Center for Sciences and Technologies for Sustainability (CCTS), campus of Sorocaba, Federal University of Sao Carlos, São Carlos 13565-905, SP, Brazil

**Keywords:** bioimaging, far-red bioluminescence, NIR bioluminescence, luciferin amino analogues, biophotonics

## Abstract

Luciferases have been extensively used as reporter genes for bioimaging purposes. For bioimaging in live animals, however, far-red- and NIR-emitting systems are better suited. Despite many efforts to develop such NIR-emitting systems, currently only a few systems have been commercialized, including AkaLuc/akalumine, which emits at ∼650 nm. Departing from the natural red-emitting luciferase of *Phrixotrix hirtus* railroadworm in combination with 6′-(1-pyrrolidinyl)­luciferin (N5), we previously reported the development of a first efficient far-red-emitting system (650 nm). Here, we further improved this system, developing a luciferase that emits far-red bioluminescence with the natural substrate d-luciferin (630 nm), and a more efficient NIR bioluminescence with 6′-amino analogues (650–664 nm). The combination of this luciferase with N5 displayed superior properties compared with the commercial akalumine/AkaLuc system (645 nm), with high affinity for N5, higher catalytic efficiency and thermostability, more sustained luminescence, and more red-shifted spectrum (660 nm). COS-1 fibroblasts expressing this luciferase/N5 combination also displayed a brighter BL signal and more red-shifted spectrum when compared with the commercial akalumine/AkaLuc system, providing a promising NIR system for mammalian BL imaging.

## Graphical abstract

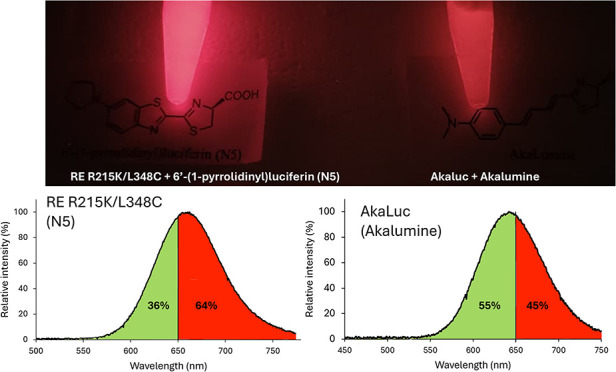

## Introduction

Luciferase and its genes have been extensively used as reporter genes for different bioanalytical applications. Since 2000, they have been increasingly used for bioimaging biological and pathological processes in mammalian animal models, including tracking metastasis, pathogenic bacteria, and viruses, helping the pharmaceutical industry to prospect new therapeutic agents.^1–6^ However, for deep tissue imaging in mammalian organisms, especially tumors, which are rich in short-wavelength-absorbing pigments such as hemoglobin, myoglobin, and melanin, red- and especially far-red-emitting luciferases are required.^7^

A lot of progresses on the comprehension of structure and function of luciferases based on the cloning of several luciferases,^8–17^ determination of the crystallographic structure,^18–21^ site-directed mutagenesis studies,^22–26^ and spectroscopic properties of luciferins and analogues have been reached, allowing to modify the protein structure of the luciferases and the chemical structure of luciferins to the advantage of specific applications.

In the case of beetle luciferases, the ones most used for bioimaging purposes since the late 1990s are red mutants, departing from firefly and click beetle luciferases.^27–32^ In the beginning of 2000, *Phrixotrix hirtus* railroadworm luciferase, the only natural red-light-emitting luciferase, also came into use.^33^ However, this luciferase had limitations such as lower thermostability and emission spectrum peaking at 623 nm with the original substrate d-luciferin. Furthermore, there are limitations to further redshift the spectrum of the wild-type and engineered beetle luciferases with the original substrate, d-luciferin, due to the structural and spectroscopic constraints of this substrate. To solve such matters, several luciferin analogues that emit red light were synthesized,^34–40^ but they also suffer from weak emission properties with the natural luciferases.

Synthesis of far-red- and NIR bioluminescence-emitting luciferin analogues has been successfully attempted.^41–45^ Among them, akalumine is the best-known example.^41^ To improve the emission properties of these analogues, some combinatorial chemistry approaches, in which the luciferase is optimized for novel red-emitting analogues, have been attempted with relative success,^41–45^ resulting in more efficient FR- and NIR-emitting systems such as akalumine/AkaLuc.^41^ However, to our understanding, these systems still emit light of intensity orders of magnitude lower than that emitted by the wild-type firefly d-luciferin–luciferase system.

Among beetle luciferases, the *P. hirtus* railroadworm is the only one that naturally produces red light (623 nm),^14^ providing a good starting point for the development of FR and NIR systems. Previous studies with large substituted 6′-amino analogues have shown that this luciferase has a larger luciferin phenolate-binding site, which accommodates well such large analogues, producing more red-shifted BL.^26^ Based on those findings, we previously developed a first mutant luciferase that produces efficient FR light (650 nm) with 6′-(1-pyrrolidinyl)­luciferin (N5) analogue.^46^ Using site-saturation mutagenesis, here we further improved this system, selectinga very efficient FR-emitting system with superior properties in relation to the commercial AkaLuc/akalumine system, which displays a brighter and more red-shifted signal suitable for mammalian cells bioimaging.

## Results and Discussion

### Background and Rationale behind Luciferase Mutant Selection

Previously, we reported the development of a *P. hirtus* mutant (R215 K) that, in combination with 6′-(1-pyrrolidinyl)­luciferin (N5), emits efficient far-red bioluminescence (650 nm) with higher thermostability in relation to the wild-type PxRE luciferase/d-luciferin combination. By using site-directed mutagenesis and 6′-substituted amino analogues displaying different sizes, including those with rings ranging from 5 to 7 atoms [piperidine (N6), 1-azepanyl (N7), and morpholine (Morpho)] (Figure 1), here we report the selection of a further improved luciferase displaying more red-shifted spectrum and higher bioluminescence activity, with superior properties in mammalian cells when compared with the commercial akalumine/AkaLuc system.

**Figure 1:**
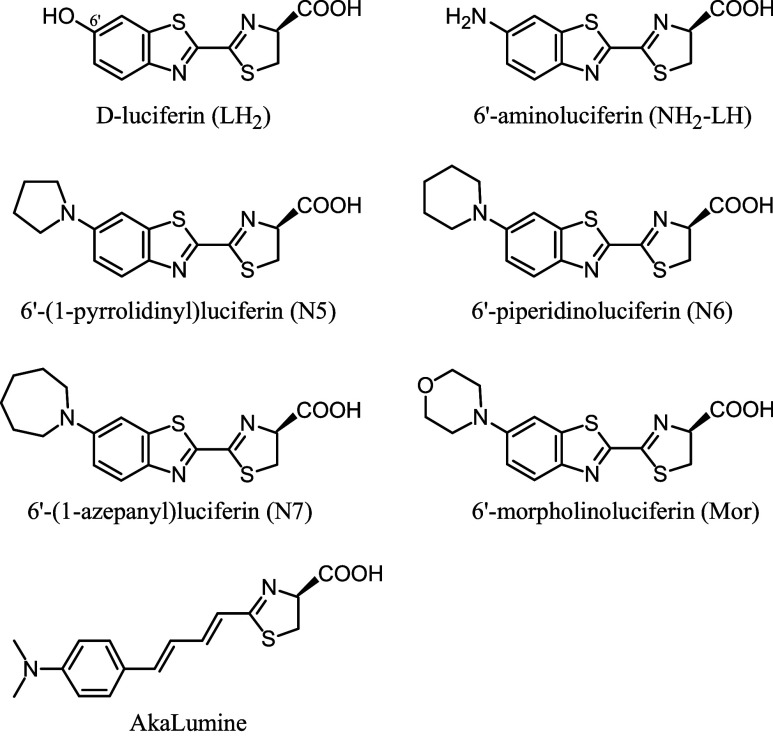
Firefly d-luciferin (LH_2_) and its analogues.

Based on previous studies by Branchini et al. with firefly luciferin^23^ and by our group with *Phrixotrix* luciferases,^24–26,46–48^ we selected some positions to perform site-directed mutagenesis with potential to improve emission properties of the R215 K *P. hirtus* red-emitting mutant luciferase. Saturating site-directed mutagenesis was performed for residues T226, H241, H242, G267, V284, C311, S314, L334, L348, N351, and R353 in search of a combination with higher activity and a more red-shifted spectrum using the 6′-(1-pyrrolidinyl)­luciferin (N5) analogue. Among the mutants, the double-mutant RE-R215 K/L348C showed the best properties.

### Bioluminescence Activity

Using d-luciferin as a substrate, the double mutant showed ∼40% of the activity in relation to the WT luciferase and 30% in relation to the single-point mutant luciferase (Table 1). However, with the N5 analogue, the bioluminescence activity was 140% higher when compared with the R215 K mutant using the same substrate and ∼160% when compared with WT luciferase with d-luciferin as substrate. Other 6′-analogues tested showed less than 40% activity in relation to d-luciferin (Figure 2).

**Figure 2:**
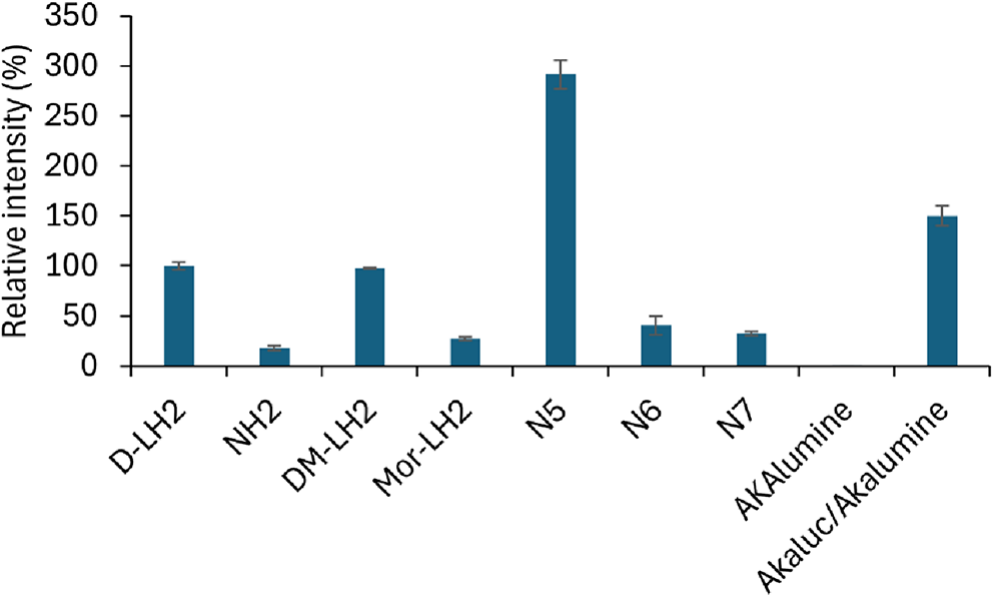
Bioluminescent activity of the R215 K/L348C mutant luciferase with different 6′-amino analogues and akalumine and AkaLuc/akalumine systems.

**Table 1:** Kinetic and Spectral Properties of Far-Red-Emitting Luciferase Combinations^a^

luciferase	Asp LH_2_ (10^9^ cps mg^‑1^)	Asp N5 (10^9^ cps mg^‑1^)	R.A^b^ LH_2_ (%)	R.A^b^ N5 (%)	*K*_M_ LH_2_ (μM)	*K*_M_ ATP (μM)	*K*_M_ N5 (μM)	*k*_cat_ LH_2_ (cps)	*k*_cat_ N5 (cps)	*k*_cat_/*K*_M_ LH_2_ (cps M^–1^)	*k*_cat_/*K*_M_ N5 (cps M^–1^)
RE-WT	70	63	100	90	7	230	0.5	8.3	7.1	1.2	14.2
RE-R215 K	89	81	127	115	40	50	1	10	9.4	0.25	9.4
RE-R215 K/L348C	30 ± 1.5	110 ± 4	57 ± 3	157 ± 6	240 ± 20	310 ± 40	5 ± 1	3.5	12.4	0.015	2.5

a Asp: specific activity.

b R.A: relative activity.

### Substrate *K*_M_ and Luminescence Kinetics

Previously, we showed that *P. hirtus* wild-type luciferase exhibits a low Michaelis–Menten constant (*K*_M_) for d-luciferin (7 μM). The red-emitting single mutant (RE-R215 K) showed more than 5-fold increase in *K*_M_ (40 μM), and the double mutant (RE-R215 K/L348C) exhibited *a* > 40-fold increase, indicating a marked reduction in affinity for the native substrate, d-luciferin. In contrast, the *K*_M_ values for the synthetic N5 analogue remained low: 0.5 μM for the wild-type enzyme, 1 μM for the single mutant, and 5 μM for the double mutant. Regarding ATP, *K*_M_ values remained high, with the double mutant displaying a *K*_M_ of 310 μM, which is even higher than that of the wild-type enzyme (230 μM).

### Catalytic Constant and Efficiencies

When the catalytic constant (*k*_cat_) of the wild-type luciferase was compared with that of the red-shifted mutants, substantial variations were observed depending on the substrate. With d-luciferin, the single mutant exhibited a ∼20% increase in catalytic activity, whereas the double mutant showed a marked reduction of approximately 50%. On the other hand, with the N5 analogue, the catalytic constant of the single and double mutants increased 30 and 75%, respectively, in relation to the wild-type enzyme. Despite these improvements in the turnover rate, both mutants exhibited elevated *K*_M_ values, which decreased the overall catalytic efficiency (*k*_cat_/*K*_M_) (Table 1). Therefore, wild-type luciferase in the presence of the N5 analogue showed the highest catalytic efficiency, primarily due to its lower *K*_M_ value, despite displaying the lowest catalytic constant. However, under substrate-saturating conditions, the higher *k*_cat_ of the double mutant compensates for its reduced efficiency, resulting in a brighter bioluminescent signal.

### Luminescence Kinetics

Kinetic analysis of the *in vitro* luminescence reaction revealed that the double mutant exhibits a slower and more sustained light emission compared to that of the single R215 K mutant for both d-luciferin and the N5 analogue. While both mutants display fast kinetics with d-luciferin, the reaction catalyzed by the double mutant proceeds at a slower rate, resulting in prolonged luminescence (with a half-life of ∼90 s). Noteworthy, with the N5 analogue, the double mutant displayed the slowest decay rate, with a half-life of 240 s, approximately 30% slower than that of the single mutant, indicating a more sustained luminescence suitable for real-time measurements (Figure 3).

**Figure 3:**
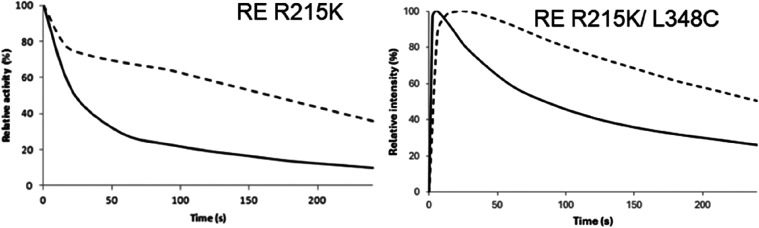
Luminescence kinetic profiles of the luminescence reaction of RE-R215 K and RE-R215 K/L348C mutant luciferases with d-luciferin (black line) and the N5 analogue (dotted line).

### Thermostability

Under the same experimental conditions, the double mutant also showed a higher thermostability in relation to the single RE-R215 K mutant luciferase, with a half-life of 20 min at 37 °C, instead of 6 min (Figure 4), providing a better-suited FR-emitting luciferase for long-time bioluminescence measurements.

**Figure 4:**
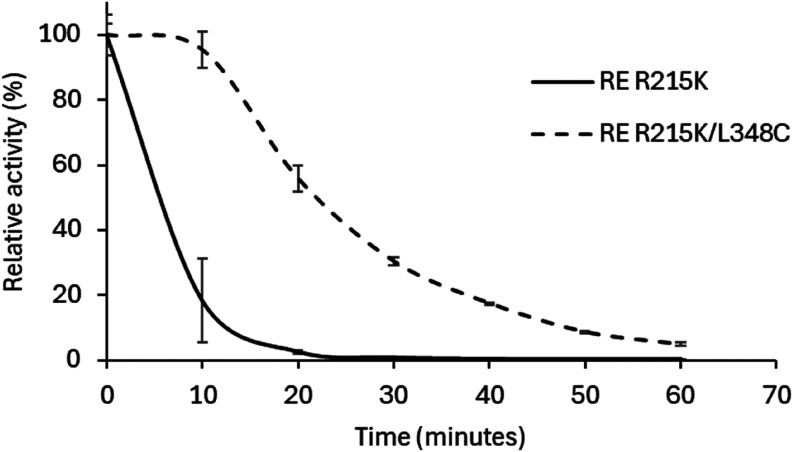
Comparison of the thermostability of RE-R215 K (black line) and RE-R215 K/L348C (dashed line) mutant luciferases at 37 °C.

### Bioluminescence Spectra

With native d-luciferin, red-shifted emissions were found for both the R215 K mutant (629 nm) and double-mutant R215 K/L348C (630 nm) luciferases. When comparing the bioluminescence spectra of these *Phrixotrix* red-emitting luciferases with 6′-amino analogues and akalumine (Table 2), the most red-shifted emission (683 nm) was obtained for the R215 K mutant with akalumine. However, the luminescence activity with akalumine was extremely low. Among the 6′-amino analogues, the most red-shifted spectra were obtained for the double mutant with the N6 and N7 analogues, with emission peaks at 662 and 664 nm, respectively (Figure 5). However, these analogues also exhibited reduced bioluminescent activity. In contrast, the N5 analogue, which displayed the highest luminescence activity among the analogues tested, also produced an additional redshift of ∼10 nm (660 nm) when compared to the single R215 K mutant luciferase (650 nm).

**Figure 5:**
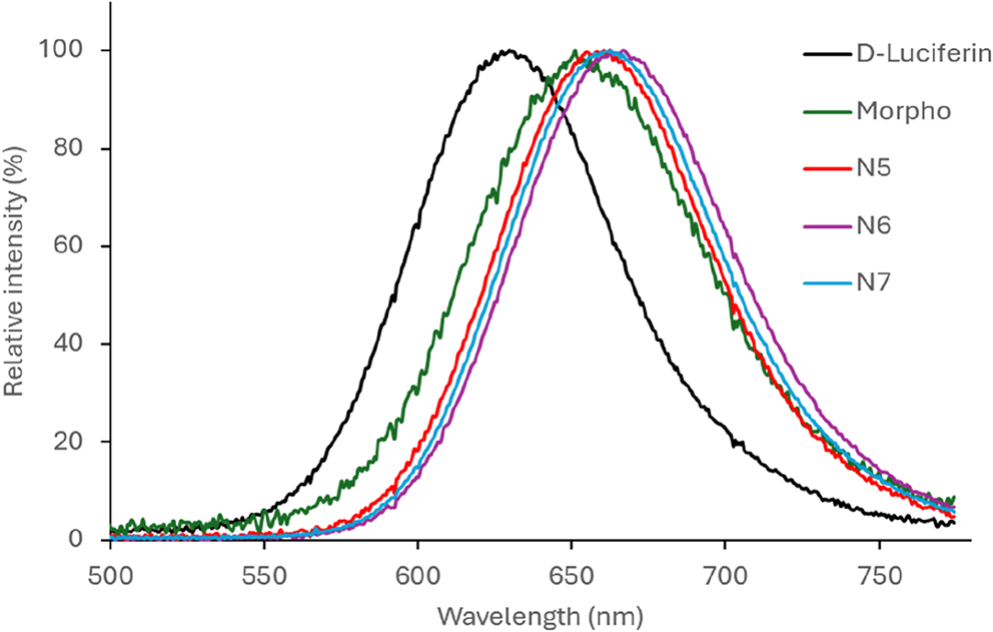
Bioluminescence spectra of the RE-R215 K/L348C mutant luciferase with different substrates.

**Table 2:** Spectral Properties of Wild-Type *P. hirtus* and Its Far-Red-Emitting Mutants with Different Luciferin Analogues

luciferase	λ_max_ (nm) LH_2_	λ_máx_ (nm) N5	λ_máx_ (nm) N6	λ_máx_ (nm) N7	λ_máx_ (nm) morpho	λ_máx_ (nm) akalumine
RE-WT	626 [82]	644 [84]	651 [84]	637 [102]	634 [92]	681 [81]
RE-R215 K	629 [77]	650 [81]	656 [76]	647 [82]	644 [72]	683 [85]
RE-R215 K/L348C	630 [79]	660 [82]	664 [83]	662 [81]	653 [88]	

### Structural Effect of the Mutation L348C

A previous work showed that the luciferin-binding pocket of *P. hirtus* red-emitting luciferase is larger than that of green-emitting luciferases, allowing this luciferase to accommodate bulky 6′-analogues, resulting in higher activity and substrate affinities and red-shifted spectra.^26^ The residue L348 is located very close to the phenolate group of oxyluciferin in the luciferin-binding site and contributes to the size of the cavity. The mutation of L348H, which decreases the size of the cavity, was shown to blueshift the spectrum.^26^ Therefore, the mutation of the larger and more hydrophobic leucine by cysteine may slightly increase the size of the substrate binding pocket, allowing better accommodation of the N5 analogue, also slightly increasing the polarity of the region, leading to a further redshift of the spectra.

### Comparison of *Phrixotrix* FR-Emitting Combinations with AkaLuc/Akalumine System

In order to compare the properties of the far-red-light-emitting luciferin–luciferase system of *P. hirtus* mutant luciferases with those of the well-established and widely used AkaLuc/akalumine system, we subcloned AkaLuc into the pCold II vector, expressed it in bacteria, purified it under the same conditions used for the *Phrixotrix* luciferases and their mutants, and compared their properties.

The double-mutant luciferase (RE-R215 K/L348C), in combination with the N5 analogue, exhibited more than twice the brightness of the AkaLuc/akalumine system under identical experimental conditions and equipment (Table 3 and Figure 2). This difference is further evidenced by the photograph of the *in vitro* bioluminescence reaction of both luciferases under identical protein and substrate concentrations (Figure 6).

**Figure 6:**
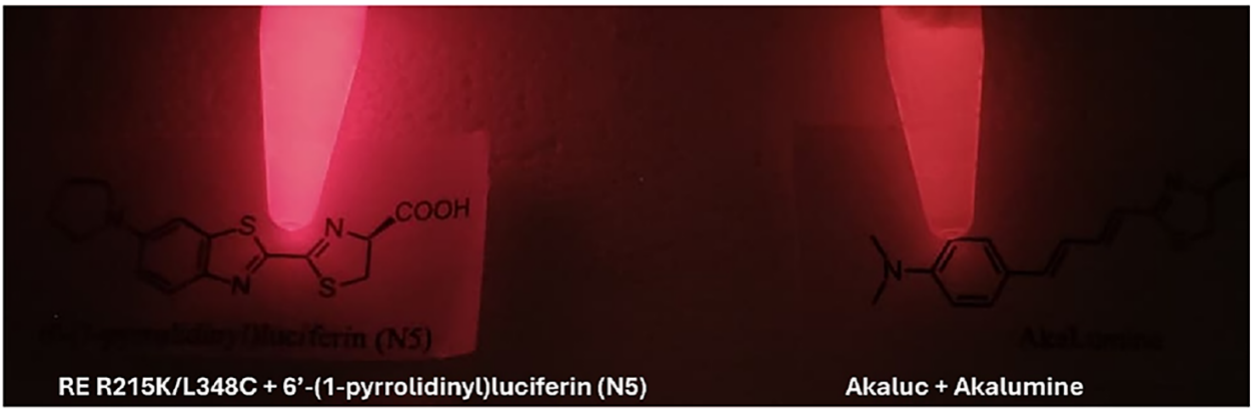
*In vitro* bioluminescence of RE-R215 K/L348C with N5 (left) and AkaLuc with akalumine (right). Photograph acquired with an Edge 20 Pro smartphone (Motorola) at ISO 3200 and 32 s exposure.

**Table 3:** Comparison of the Bioluminescent Activities of FR-Emitting Combinations.

luciferase	specific activity LH_2_ (10^9^ cps mg^‑1^)	specific activity N5 (10^9^ cps mg^‑1^)	specific activity akalumine (10^9^ cps mg^‑1^)
RE-WT	70	63	0.9
RE-R215 K	89	81	0.4
RE-R215 K/L348C	30 ± 1.5	110 ± 4	
AkaLuc	3.5 ± 0.5	7.8 ± 1	45 ± 3

We also compared the *in vitro* bioluminescence spectra of the *Phrixotrix* luciferase combinations and commercial akalumine/AkaLuc. Noteworthy, the combination of the single and double *Phrixotrix* luciferase mutants with the N5 analogue displayed more red-shifted spectra in relation to the AkaLuc/akalumine system under the same experimental conditions (Figure 7).

**Figure 7:**
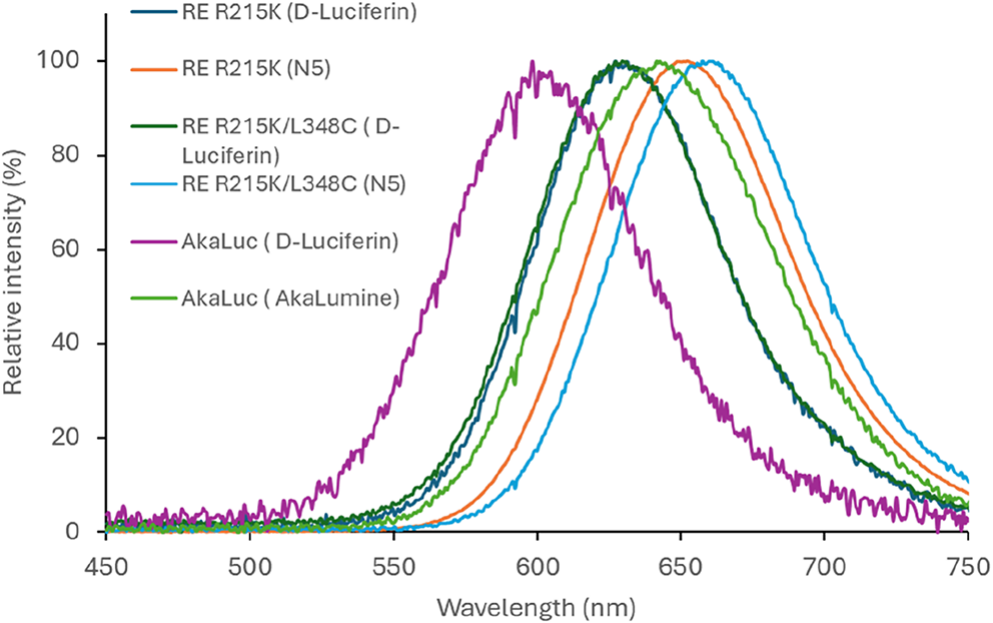
Comparison of the bioluminescence spectra of different far-red-emitting systems.

Furthermore, in order to compare the proportion of emitted light in the far-red region by the *Phrixotrix* double mutant/N5 and AkaLuc/akalumine combinations, we calculated the fraction of light emitted above 650 nm in the bioluminescence emission spectra of these combinations. The *P. hirtus* mutant exhibited ∼65% of its emission above 650 nm, whereas the AkaLuc/akalumine system showed 45% above 650 nm, indicating a higher proportion of NIR light in the former system and, consequently, a greater potential for deep tissue penetrability in mammalians (Figure 8).

**Figure 8:**
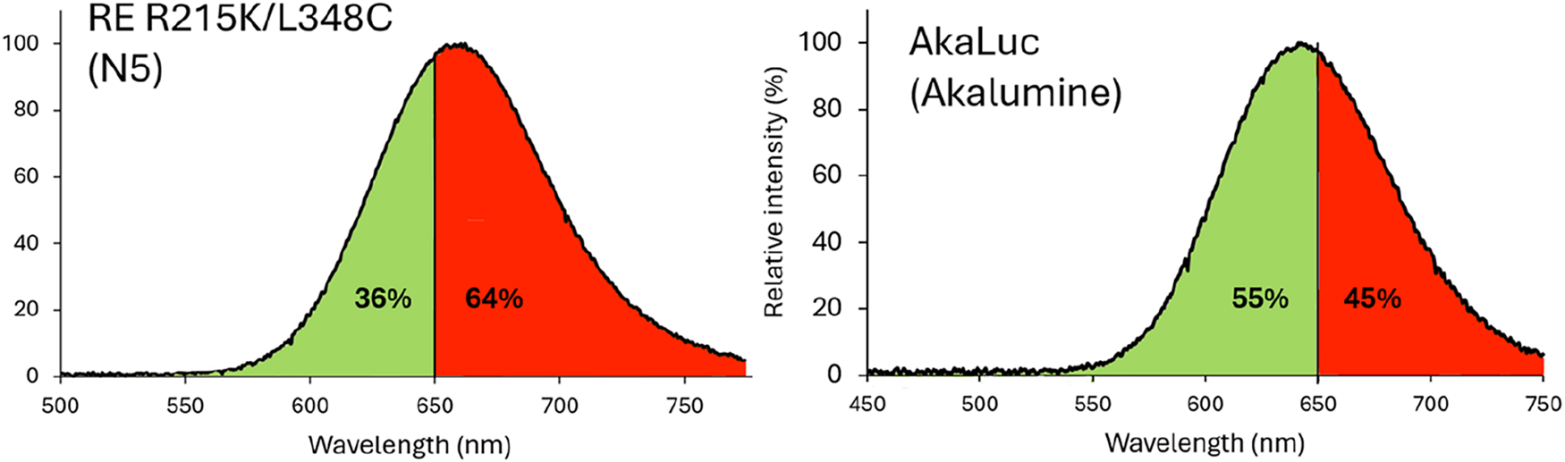
Proportion of Far-Red light emission in the bioluminescence spectra of RE-R215 K/L348C mutant luciferase with N5 (left) and AkaLuc with akalumine (right). The green area corresponds to wavelengths below 650 nm, and the red area corresponds to those above.

### Comparison of *Phrixotrix* Luciferase/N5 and Akalumine/AkaLuc in Mammalian Cells

We also compared the BL activity of COS-1 cells transfected with plasmid pCMV containing RE-R215 K, the double mutant, and AkaLuc, with their respective substrates. Among all luciferases, the double mutant/N5 combination showed the highest initial *in vivo* BL activity, followed by the RE-R215 K/N5 and akalumine/AkaLuc systems (Figure 9). The kinetic profiles of these combinations were generally similar, with an initial peak followed by sustained luminescence for several hours. Notably, whereas during the first hour of measurement, the *P. hirtus* mutant/N5 combination displayed a higher initial activity that decayed and stabilized after approximately 30 min, the AkaLuc/akalumine system displayed a slower kinetics, reaching a similar maximum activity to the *P. hirtus* mutant/N5 system only after 90 min (Figure 10). Such a different kinetic profiles may reflect the better membrane permeability of the N5 analogue, which is more hydrophobic, resulting in a higher initial bioluminescence signal after addition to the cells. Similar to the *in vitro* reaction, the double mutant/N5 also showed the most red-shifted spectrum in mammalian cells when compared to AkaLuc/akalumine (Figure 11).

**Figure 9:**
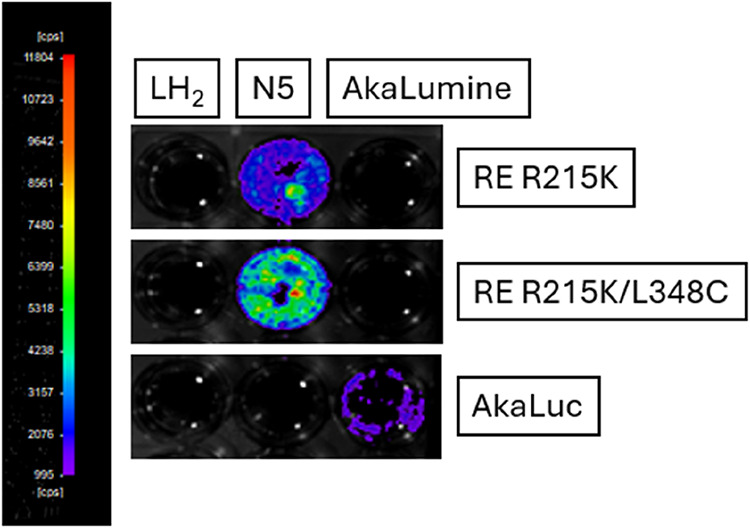
Comparison of the initial bioluminescence of COS-1 transfected with plasmids harboring the cDNA of RE-R215 K, RE-R215 K/L348C, and AkaLuc just after the addition of different substrates.

**Figure 10:**
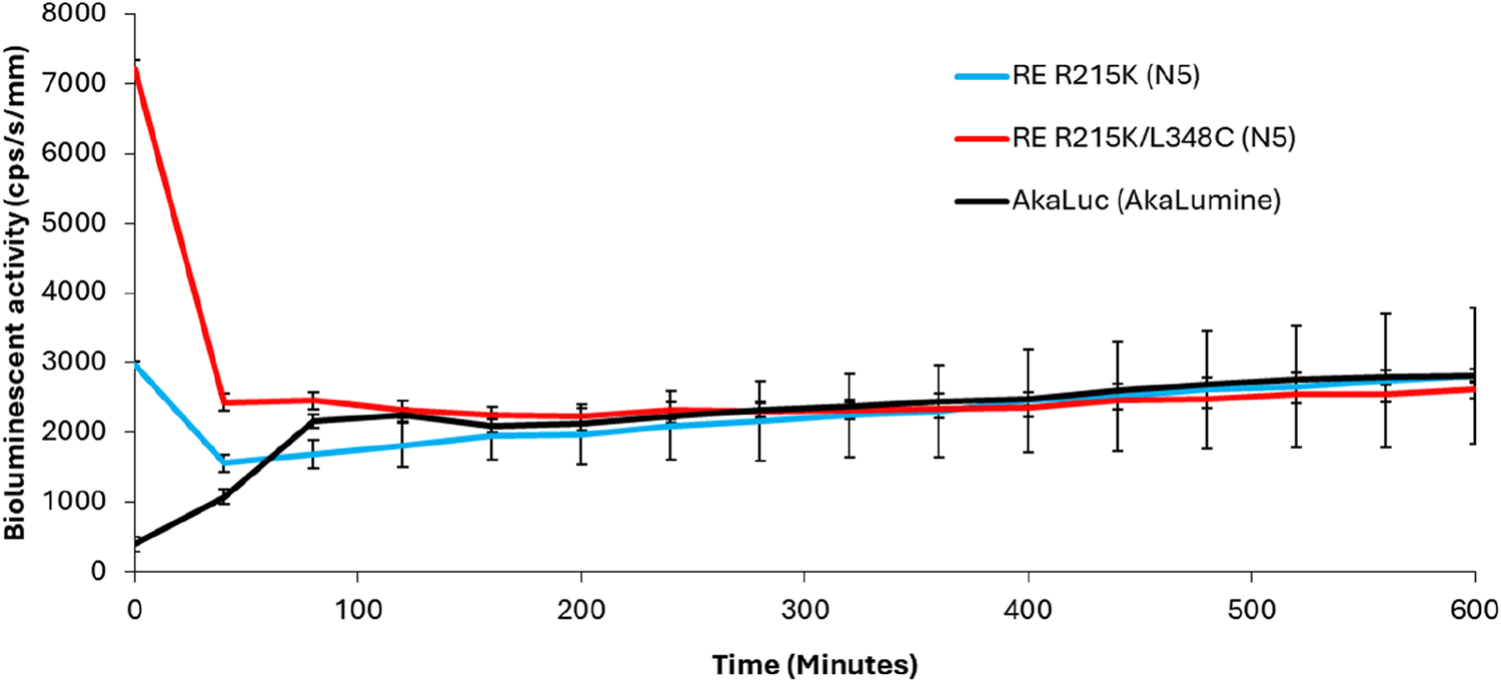
Kinetic profiles of the *in vivo* bioluminescence of COS-1 cells transfected with plasmids harboring the cDNAs of RE-R215 K, RE-R215 K/L348C, and AkaLuc luciferases, after the addition of different substrates.

**Figure 11:**
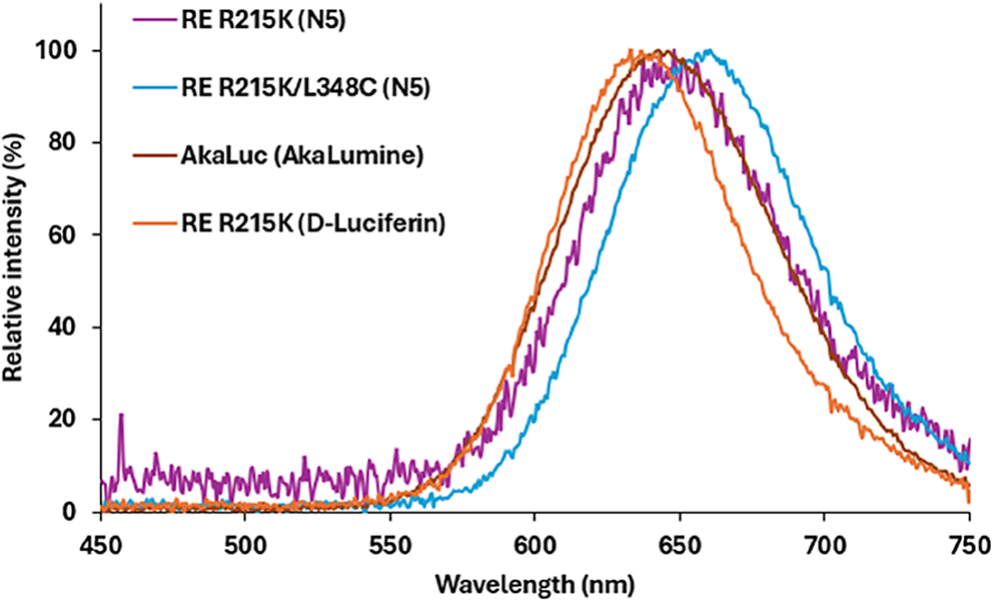
Bioluminescence spectra of COS-1 cells transfected with plasmids harboring the cDNAs of RE-R215 K, RE-R215 K/L348C, and AkaLuc luciferases after the addition of different substrates.

## Concluding Remarks

Here, we introduce an improved far-red-emitting luciferin–luciferase system developed from the natural red-emitting luciferase from *P. hirtus* railroadworm in combination with the 6′-(1-pyrrolidinyl)­luciferin analogue. This combination exhibits higher bioluminescence activity, the most red-shifted spectrum, improved stability, and more sustained kinetics when compared to the previous RE-R215K/N5 and commercial AkaLuc/akalumine combinations. Furthermore, this mutant RE-R215 K/L348C luciferase combination also displayed higher luminescence activity and the most red-shifted spectrum in mammalian cells, providing a new, highly promising luciferin–luciferase combination for deep tissue bioimaging purposes.

## Materials and Methods

### Plasmids and Beetle Luciferase cDNAs

The cDNAs for *P. hirtus* red-emitting (PxRE) and its mutant R215 K inside the pCold vector (Takara) were obtained from the previous work.^46^ Commercial plasmid containing AkaLuc luciferase was purchased from Addgene (Plasmid #120371).

### cDNA Subcloning

The cDNA of the far-red-emitting mutant of *P. hirtus* (RE-R215 K) was amplified by PCR and subcloned into *Nde* I and *Hind* III restriction sites of the pcDNA1 vector (Invitrogen) to produce pCMV-RE-R215 K. The cDNA of the commercial far-red-emitting luciferase AkaLuc was amplified by PCR and subcloned into *Nde* I and *Hind* III restriction sites of the pCold II (Takara, Japan) to produce pC-AkaLuc.

### Site-Saturation Mutagenesis

The mutants of RE-R215 K luciferase were obtained by site-directed mutagenesis using a Thermo Fisher Scientific kit (catalogs F-530XL and F530L). The plasmids containing the luciferase cDNAs were amplified using *Phusion* polymerase and 2 complementary *primers* containing the degenerated codon in the 348 position, using a thermal cycler (1 cycle 98 °C, 2 min; 25 cycles 98 °C, 30 s; 55 °C, 1 min and 72 °C, 6 min). After amplification, mutated plasmids containing staggered nicks were generated. The products were treated with *Dpn* I in order to digest nonmutated parental plasmids and used directly to transform *Escherichia coli* XL1-Blue cells (Agilent). The following forward and respective reverse primers were used (the mutation codon is highlighted in bold): RE-L348X forward: GCCCTGATC**NNS**AGCCCCAACG and reverse: CGTTGGGGCT**SNN**GA TCAGGGC.

### Luciferase Expression and Purification

For luciferase expression, transformed *E. coli* BL21-DE3 cells were grown in 100–1000 mL of the LB medium at 37 °C up to OD_600_= 0.4 and then induced at 18 °C with 0.4 mM IPTG for 18 h. Cells were harvested by centrifugation at 2500*g* for 15 min and resuspended in extraction buffer consisting of 0.10 M sodium phosphate buffer, 1 mM EDTA, 1 mM DTT, 1% Triton X-100, 10% glycerol, protease inhibitor cocktail (Roche), pH 8.0, lysed by ultrasonication, and centrifuged at 15,000*g* for 15 min at 4 °C. The N-terminal histidine-tagged luciferases were further purified by agarose-nickel affinity chromatography (QIAGEN), using the following buffers: (wash buffer) 50 mM phosphate pH 7.0, 300 mM NaCl, and 20 mM imidazole; (elution buffer) 50 mM phosphate pH 7.0, 300 mM NaCl, and 250 mM imidazole; and (dialysis buffer) 25 mM Tris–HCl pH 8.0, 10 mM NaCl, 1 mM EDTA, 2 mM DTT, and 10% glycerol. The concentrations of purified luciferases were between 0.2 and 0.5 mg/mL, and the estimated purity, according to SDS-PAGE gels, was about 90% (Figure S1).

### Measurement of *In Vitro* Luciferase Activity

Luciferase bioluminescence intensities were measured using an AB2200 (ATTO; Tokyo, Japan) luminometer. The assays were performed by mixing 5 μL of 40 mM ATP/80 mM MgSO_4_ with a solution consisting of 5 μL of purified luciferase, 5 μL of 10 mM d-luciferin or 2 mM luciferin analogue, and 85 μL of 0.1 M Tris–HCl pH 8.0 at 22 °C. All measurements were done in triplicate for at least three independent luciferase preparations, and the averages and the standard deviations are reported in the figures.

### Thermostability

The purified *P. hirtus* luciferase mutants were incubated in a dialysis buffer (25 mM Tris–HCl, pH 8.0, 10 mM NaCl, 1 mM EDTA, 2 mM DTT, and 10% glycerol) at 37 °C, and the bioluminescent activity was measured at various time intervals.

### Kinetics Measurements and *K*_M_ Determination

The *K*_M_ assays for luciferin were performed by mixing 5 μL of 40 mM ATP, 80 mM MgSO_4_ in a solution containing 10 μL of luciferase, 75 μL of 0.10 M Tris–HCl (pH 8.0), and luciferin and N5 at final concentrations between 0.01 and 1 mM. The *K*_M_ assays for ATP were performed by mixing 5 μL of 80 mM MgSO_4_ in a solution containing 10 μL of luciferase, 75 μL of 0.10 M Tris–HCl (pH 8.0), and ATP at final concentrations of 0.02–2 mM. Both assays were performed in triplicate. The *K*_M_ values were calculated using Lineweaver–Burk plots, taking the peak of intensity (*I*_0_) as a measure of *V*_0_. All measurements were performed in triplicate of three independent luciferase preparations, and averages with standard deviations were reported. The kinetics of the luminescence reaction were recorded using a TD-III luminometer (Japan). In the assay tube, 5 μL of a solution consisting of 80 mM MgSO_4_ and 40 mM ATP was mixed with a solution consisting of 10 μL of luciferase (∼0.2 mg/mL) and 10 mM LH_2_ in 75 μL of 0.10 M Tris–HCl buffer of pH 8.0.

### Determination of *k*_cat_ and *k*_ox_

The overall *k*_cat_ and *k*_ox_ were determined according to eq 2 (Supporting Information) by calculating the ratio of luminescence activities in *cps* by the number of luciferase molecules based on the specific bioluminescence activities measured with luciferin and ATP (overall *k*_cat_) and with luciferyl-adenylate (*k*_ox_), respectively. Because the absolute value of *cps* in photons/s could not be determined, the absolute values of *k*_cat_ and *k*_ox_ in s^–1^ could not be determined; therefore, the values were reported in cps (counts per second). The activities were normalized by the spectral sensitivity of the AB2200 luminometer (ATTO; Tokyo, Japan). Although these values are not absolute, they can be safely used as relative values of catalytic constants.

### 6′-Substituted Amino Analogues

All the 6′-substituted aminoluciferin analogues (Figure 1) were synthesized as previously reported.^35^ Stock solutions of 10 mM were prepared in DMSO and kept at −20 °C in the dark. Akalumine luciferin analogue was purchased from Sigma-Aldrich.

### Bioluminescence Spectra

The bioluminescence spectra reported here were recorded using an ATTO Lumispectra spectroluminometer (Tokyo, Japan) with a cooled CCD camera. For the *in vitro* bioluminescence recorded using the spectroluminometer, 5.0 μL of luciferases were mixed with 90 μL of 0.10 M Tris–HCl of pH 8.0, 5 μL of specific substrate (10 mM d-luciferin; luciferyl-adenylate or 6′-aminoluciferin analogues), and 5 μL of 40 mM ATP/80 mM MgSO_4_. The bioluminescence spectra were measured in triplicate for at least three independent luciferase preparations, and the reported spectra represent the average. The peaks were manually estimated, and the peak variation was ±2.5 nm. Above 620 nm, due to the lower energy above this wavelength, we assumed peak errors of ±3 nm. The backgrounds were automatically subtracted from each spectrum. The fraction of emitted light in the spectrum above and below 650 nm was calculated by the ratio between the sums of the counts in each spectral region (550–650 and 650–750).

### Fibroblast Culture and Transfection

An amount of 2.5 × 10^5^ COS-1 cells was previously seeded in a 6-well plate, or 5 × 10^4^ cells in a 24-well plate, depending on the experiment. The cells were kept in DMEM at a high concentration of glucose with l-glutamine and phenol red (DMEM) supplemented with 10% fetal bovine serum (Cultilab, Campinas, SP, Brazil) and 1% penicillin/streptomycin/amphotericin in an incubator with a humidified 5% CO_2_ atmosphere at 37 °C. COS-1 cells were acquired from Rio de Janeiro Cell Bank (BCRJ, cell type code 070 batch number 000114). The COS-1 cells were transfected each time with the same amounts of plasmids, ranging from 0.5 to 1.5 μg pcDNA1-RE-R215 K, pcDNA1-RE-R215 K/L348C, and N1-AkaLuc/well, using the Lipofectamine 3000 (Invitrogen-Thermo Fisher, São Paulo, Brazil) reagent following the manufacturer’s instructions.

### Comparison of *In Vivo* Bioluminescence in Transfected Fibroblasts

During the second day after the transfection, we added 10 μL of 100 mM d-luciferin or 10 mM luciferin analogue or 10 mM akalumine to adhered confluent transfected COS-1 cells and measured their bioluminescent activity (cps) for 30 s, using a multiusuary NightOwl Bioluminescence Photodetection Camera system (Berthold Technologies GmbH & Co. KG, Bad Wildbad, Germany). The bioluminescent activity was monitored using the same equipment at time intervals of 40 min between the images for a period of 12 h. The graph of activity versus time was plotted as a function of the relative activity of the luciferases. The experiments were repeated 5 times.

## Supplementary Material



## Data Availability

All data are available in the manuscript, and additional information can be obtained upon request.

## Notes

SDS-PAGE of purified proteins (PDF)
