# Supplementary material for: Brighter NIR Bioluminescence System for Mammalian Cell Bioimaging Based on Engineered Railroadworm Luciferase and 6′-Aminoluciferin Analogues
Source: Chem Biomed Imaging. 2026 Jan 14;4(6):1098–106. doi: 10.1021/cbmi.5c00163 (PMC13291975; doi:10.1021/cbmi.5c00163)
Supplement: Supplementary file 1 [file im5c00163_si_001.pdf]

Supporting Information

A brighter NIR bioluminescence system for mammalian cell bioimaging based on engineered railroadworm luciferase and 6´amino-luciferin analogs

Gabriel F. Pelentir [1], Vanessa R. Bevilaqua [2], Michio Kakiuchi[3], Takashi Hirano [3] and Vadim R. Viviani [1]*

[1] Graduate Program of Biotechnology, Federal University of São Carlos (UFSCar), São Carlos, SP, Brazil.

[2] Biomaterials Laboratory, Medical and Health Sciences Faculty, Pontifical University Catholic of São Paulo (PUC-SP), Sorocaba 18060-030, SP, Brazil.

[3] Department of Engineering Science, Graduate School of Informatics and Engineering, The University of Electro-Communications, Chofu, Tokyo, 182-8585, Japan

Corresponding Author

Vadim R. Viviani - Department of Physics, Chemistry and Mathematics, Graduate Program of Biotechnology and Environmental Monitoring, Federal University of São Carlos, Rod. João Leme dos Santos, Km 110, Sorocaba (SP), Brazil, Telephone: (55) 015 3229-7514, *E-mail:* viviani@ufscar.br

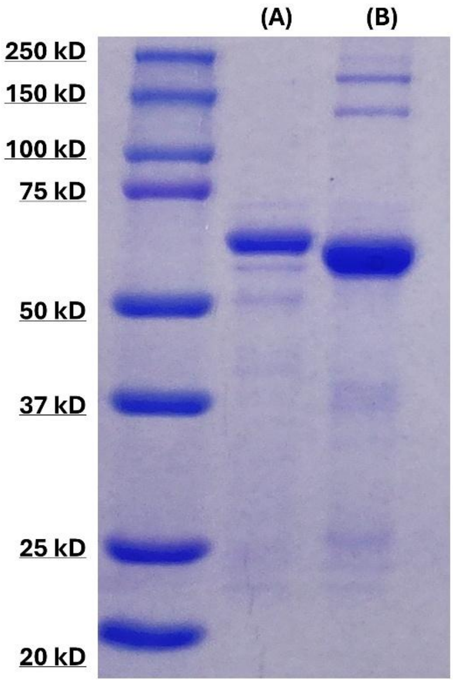

Figure S1. SDS-PAGE of purified AKALuc (A) and RE R215K/L348C (B) luciferases.
